# Giant Complex Odontoma of Mandible: A Spectacular Case Report

**DOI:** 10.2174/1874210601711010413

**Published:** 2017-06-30

**Authors:** Narjiss Akerzoul, Saliha Chbicheb, Wafaa El Wady

**Affiliations:** Department Of Oral Surgery, C.C.D.T, Faculty Of Dentistry, University Mohamed V, Rabat, Morocco

**Keywords:** Complex odontoma, Mandible, Odontogenic tumors, Hamartomas, Benign tumors

## Abstract

**Introduction:**

Odontomas are considered as benign tumors of odontogenic tissue origin and are more over non-aggressive. They can also be categorized as hamartomas and are a result of developmental malformation of odontogenic tissues. As the name suggests, they are composed of mature tooth substances. They possess limited and slow growth potential and are well differentiated. They can be ectodermal, mesodermal or mixed in origin. Mixed variety may be further divided into compound or complex depending upon their radio-graphical resemblance to the tooth. Compound odontomes are reported to be twice more common than complex odontomes. Among them, complex odontomes are asymptomatic unless they cause bony expansion of the jaws.

**Case Report:**

This paper aims to report and discuss a case of complex odontoma with unusually large size leading to gross facial asymmetry. Further this paper will highlight the important information the general dental practitioner must possess to diagnose such lesions at an early stage.

**Conclusion:**

Odontomas are benign odontogenic tumors with unusually large size leading to gross facial asymmetry. The general dental practitioners must possess the knowledge and important information to diagnose such lesions at an early stage.

## INTRODUCTION

Odontomas are benign in nature and arise from the odontogenic tissues [1-15]. These are reported to be developmental in origin [1-6, 16-21]. Due to their origin from the well differentiated cells of odontogenic epithelium namely odontoblasts and ameloblasts, they are radiographically and histologically characterized by the production of mature enamel, dentin, cementum as well as pulp [1-11, 15, 16]. It is further evidenced that this production of enamel, dentin, cementum as well as pulp is in abnormal fashion [15].

It was in 1947 when the term Odontoma was coined by Paul Broca [11, 20, 21]. The etiology of these tumours is unknown although some of the authors reveal it to be genetic in origin, or due to trauma or infection [15-20]. There is no gender predisposition [12-20]. Clinically these tumors are asymptomatic and non-aggressive and may present with missing teeth from the jaw [14, 15]. While they may present as facial asymmetry in case they achieve a big size [1-11].

Several authors have classified odontomas into various varieties [15, 16]. WHO in 2005 classified odontomas in two types namely compound and complex odontomas [12, 13, 19]. Literature reveals that they can also be termed as central, peripheral and erupted Odontoma [12, 13, 19, 22-30]. The central odontomas are present well inside the jaw bones while the peripheral one presents in the soft tissue over the alveolar bone where the tooth is impregnated. Radiographically, the complex odontomes will present as radiopaque or calcified dental tissues which are irregular in shape. Moreover the mass will not carry any morphological similarity to the teeth [15, 16].

Erupting odontom is the one in which the hemartomatous mass is visible inside the oral cavity clinically [1, 12, 13, 19].

Although odontomes are a benign entity and can be easily diagnosed, the dental professionals must be well versed with the clinical presentation as well as the radiological presentation of the same. Sometimes, the lesion attains an unusual large size [22-26]. Henceforth, the aim of this paper was to describe a case of giant complex odontoma in a 35 year old male, located at the mandibular angle. This paper also highlights the clinical as well as radiological aspects of the disease along with the surgical management.

## CASE REPORT

A 35 years old male patient visited the department of Oral Surgery of the Consultation Center of Dental Treatment (CCDT) of Rabat with a swelling on the left side of the mandible. The swelling was painless and was present since last 2 weeks. His medical history was not significant. Clinically, the patient presented with asymmetry of the face. There was a diffuse bony hard swelling present on the mandibular angle region of the left side. On the contrary, the overlying skin was normal with no tenderness with normal mouth opening.

**Fig. (1):**
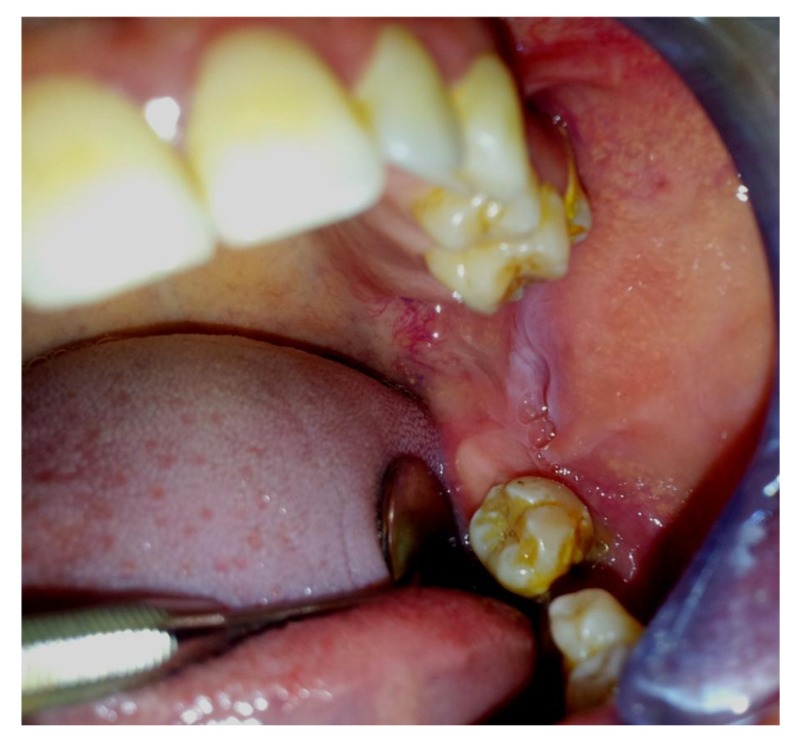
Pre-operative View.

**Fig. (2):**
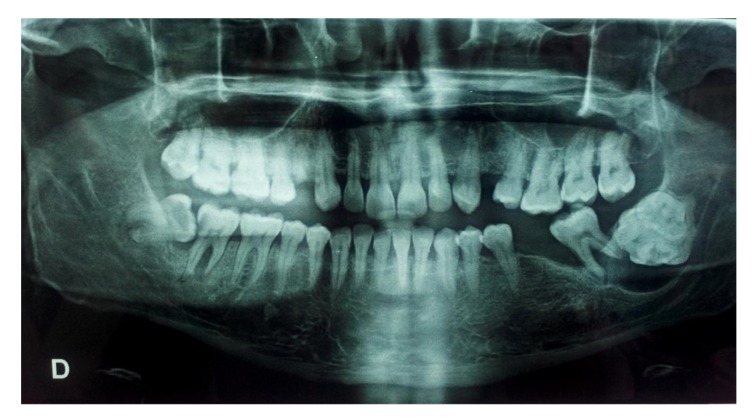
Panoramic radiograph showing the lesion as well defined radiopacity in the left side of the mandible in the angle region.

**Fig. (3):**
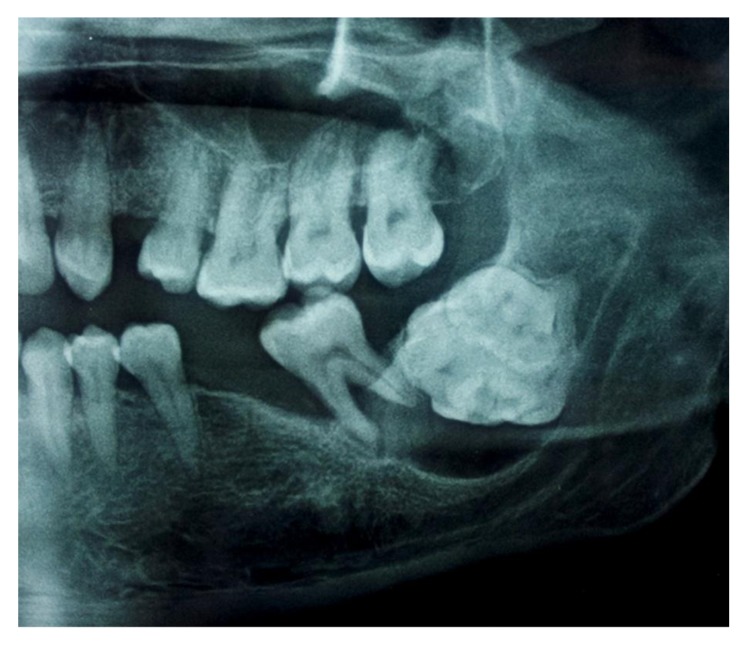
Panoramic radiograph revealing a well defined radiopacity in the left side of the mandible in the angle region with the secondary inferior displacement of the mandibular canal w.r.t 37.

Intraorally, the patient presented with missing left first and third molar along with a breach in the corresponding alveolar mucosa in relation to 38 region (Fig. **1**). There was evidence of bucco-lingual expansion of the mandible in the same region which was hard and non tender on palpation. Further there was no significant lymphadenopathy. Provisional diagnosis considered was impacted by third molar with suspected dentigerous cyst. An Orthopantomogram was taken (Fig. **2**, **3**).

**Fig. (5):**
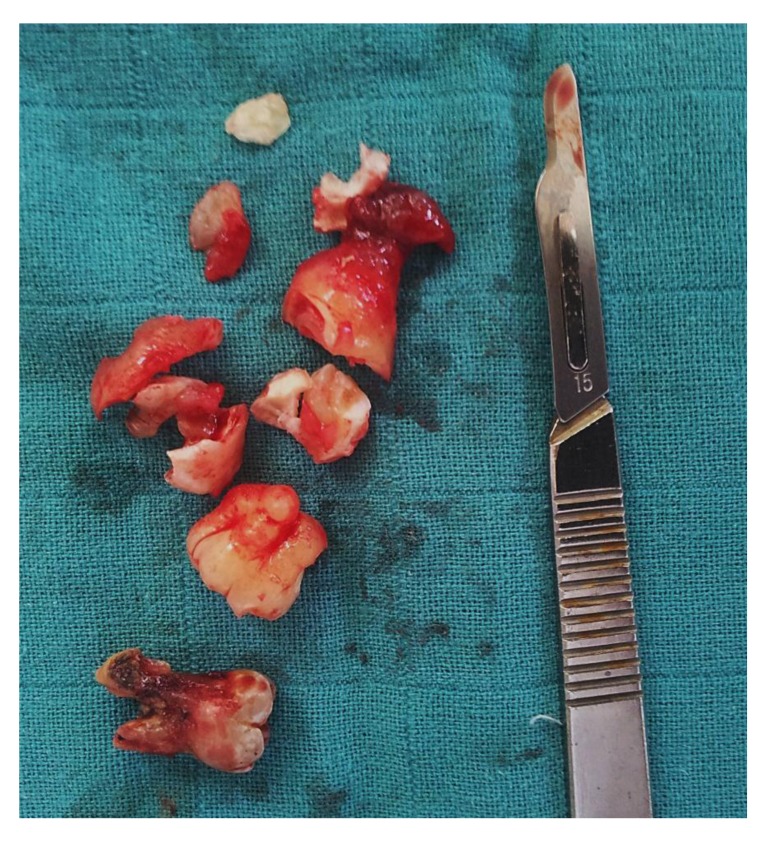
Excised lesion.

**Fig. (6):**
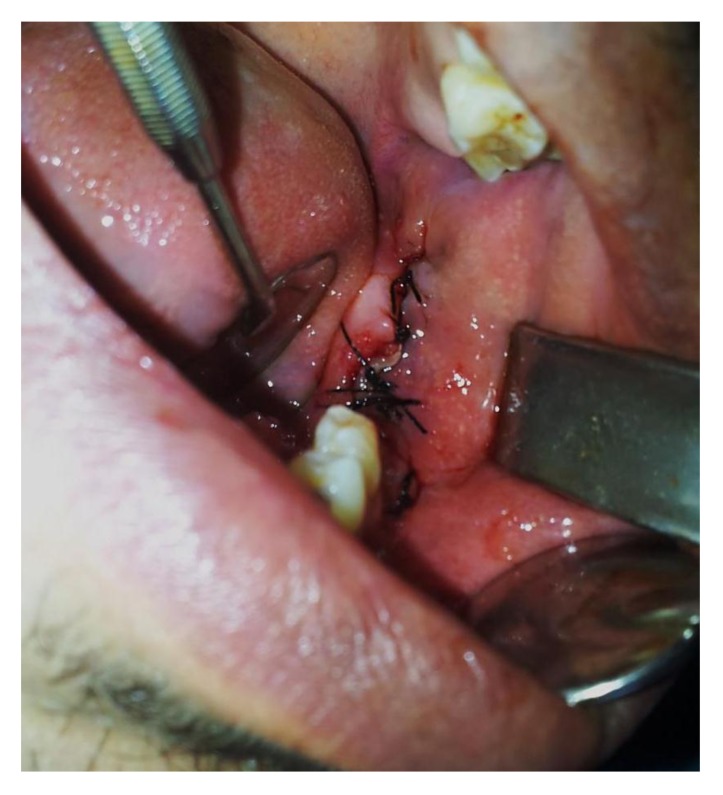
Post-operative view with sutures.

The Orthopantomogram (OPG) revealed a spectacular well-defined radiopacity, of about 6 cm x 6 cm in dimension, surrounded by a radiolucent halo with corticated margin approaching the apical region of 37. Further there was evidence of secondary inferior displacement of inferior alveolar nerve canal around the 37 (Figs. **2**-**3**). The clinical and radiographic presentation of the lesion led to a diagnosis of complex odontoma. Under loco-regional anesthesia, the surgery was performed, consisting of the excision of the whole impacted teeth and the lesion all around Fig. (**4**). The excised lesion specimen (Fig. **5**) was sent to anatomopathological study which concluded a complex odontoma. The histological examination was also done which revealed that the lesion was encapsulated with evidence of cementum and dentine like structures along with pulpal tissue and epithelial remnants. The patient was followed-up over a period of one month (Fig. **6**).

## DISCUSSION

Odontomas are the most commonly occurring odontogenic tumors with an incidence rate of 22% [2-10]. Further literature supports them of having a developmental nature as most of them start developing along with the development of normal dentition [1-8]. Although the odontomes can occur anywhere in the jaws, compound odontomes occur most frequently in the canine and incisor region of the maxillary arch [14, 15]. On the contrary, the complex odontomes are found more commonly at the mandibular molar region [3, 12, 13, 19].

As discussed earlier, complex odontomas are usually asymptomatic [25-30]. Further, it is also evidenced that most of the time they are of small size, rarely exceeding the size of a tooth with which they are associated [7, 14, 15]. Sometimes they become unusually large in size as presented in this case. In such cases, they present with alveolar swelling in the jaw leading to facial asymmetry and expansion of the cortical plates. The tooth will be unerupted or missing clinically [15, 16]. It can also lead to malpositioning, deviation or impaction of the adjacent teeth [4, 5, 15, 16]. This was also seen in this present case described.

Literature also provides an evidence of eruption of odontoma in the oral cavity at the alveolar arch which is often painful and can subsequently lead to inflammation of adjacent soft tissues [12-20]. At this moment, it may also be confused with some bony lesion [4, 5, 12, 13, 19]. Since most of the odontomas are asymptomatic, henceforth they are detected during routine dental examination. The most common age of occurrence is second decade *i.e.* 12-18 years [16, 17].

As described earlier, the odontomas may be central (intraosseous) or pheripheral (extraosseous) [5-15, 25]. The present case is a case of intraosseous odontoma which occur totally inside the jaws leading to expansion of the bone. The third variety of odomtoma is erupted odontoma [17, 18]. The procedure of eruption of such odontomas in the oral cavity is different as compared to the eruption pattern of normal teeth. This is attributed to the absence of periodontal ligament fibers in odontoma [7-12]. In the present case, although the odontoma was intraosseous, yet it presented with breach in the alveolar mucosa. in the region of the lesion. This may signal towards the progressive eruption of the odontoma into the oral cavity [25-30].

Certain authors are of the view that since the complex odontome is associated with unerupted tooth, hence it is the eruptive force of the concerned uneruptive tooth that can lead to eruption of the odontoma in the oral cavity [7, 10-12, 22]. This theory of odontoma eruption may suit to this presented case too as in this case, the odontoma was present coronal to the lesion. Further it may also be due to the resorption of the bone evidenced on the radiograph [8-15]. Further, odontoma may also lead to bone resorption in case the lesion is increasing in size [5]. Certain authors like Ragallli *et al.* were of the view that it is the growth of capsule that contributes to the eruption of odontoma [8, 23-25]. The literature reveals that the preferred treatment plan for such lesions is the enucleation of the lesion [15-18]. This will let the impacted teeth to erupt in the oral cavity [26, 27]. On the contrary in this reported case, since the lesion was bordered by a radiolocency suspected to be dentiogerous cyst, the treatment included removal of the tooth along with the lesion [28-30].

## CONCLUSION

Complex odontomas are odontogenic tumors and are asymptomatic in nature. They are usually found during routine dental radiographic examination and are not associated with any other systemic disease. They can even erupt inside the oral cavity and can lead to pain and inflammation. Large lesions can cause expansion of the cortical plates of the jaws leading to facial asymmetry. Henceforth, the dental professionals must be well aware of the clinical as well as the radiological findings of such lesion so as to provide prompt treatment with better prognosis.

## Figures and Tables

**Fig. (4):**
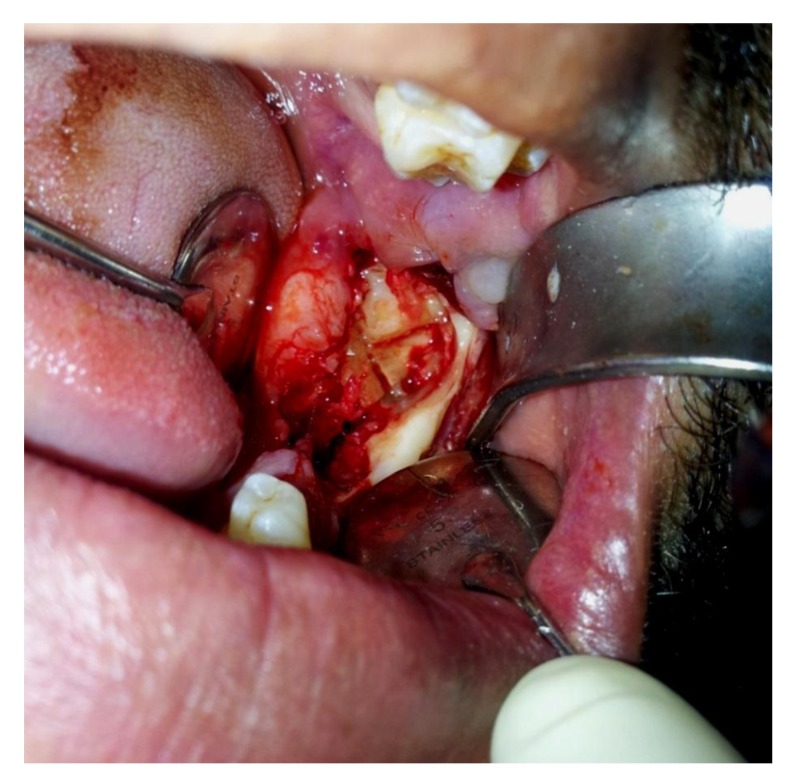
Surgical Exposure of the tumor.
